# Heterogeneous Fenton Degradation of Patulin in Apple Juice Using Carbon-Encapsulated Nano Zero-Valent Iron (CE-nZVI)

**DOI:** 10.3390/foods9050674

**Published:** 2020-05-24

**Authors:** Notemba Silwana, Blanca Calderón, Seteno Karabo Obed Ntwampe, Andrés Fullana

**Affiliations:** 1 University Institute of Water and Environmental Sciences, University of Alicante, San Vicente del Raspeig Road, San Vicente del Raspeig, Alicante 03690, Spain; blanca.calderon@ua.es (B.C.); andres.fullana@ua.es (A.F.); 2 School of Chemical and Minerals Engineering, North West University, Private Bag X1290, Potchefstroom 2250, South Africa; Karabo.Ntwampe@nwu.ac.za

**Keywords:** apple juice, heterogeneous Fenton oxidation, nano-zerovalent iron particles, patulin degradation

## Abstract

Patulin (PAT), a mycotoxin found mainly in matured apples, is produced by different species of fungi, mainly *Penicillium expansum,* and is found in various fruits and vegetables used to produce juice. Little focus has been placed on nano-technological methods for the mitigation of this problem. In this work, carbon-encapsulated nano-zero valent iron (CE-nZVI) particles were synthesized and used as heterogeneous Fenton agents for the degradation of PAT in apple juice. The particles were found to have a spherical shape with a diameter of 130 ± 50 nm. In a heterogeneous Fenton degradation (involving CE-nZVI) process, a concentration of 0.05 g/L CE-nZVI with 0.5 mM H_2_O_2_ was used. Since the Fenton oxidation process is pH-dependent, placebo degradation was observed at varying pH conditions with an average percentage of PAT degradation of 27.8%, 87.0%, 98.0%, and 99.75% at pH 6, 5, 4.5, and 3.5 respectively, between 1 min to 4 h in a water matrix. In a juice matrix, at the regular pH of juice (3.6), percentage PAT degradation of 72% and 89% was obtained after a 2-h treatment using heterogeneous Fenton oxidation (CE-nZVI/H_2_O_2_) systems, using 0.5 mM H_2_O_2_ and 1 mM H_2_O_2_, respectively.

## 1. Introduction

Apple cultivators and apple-derived product producers are faced with an enormous challenge, i.e., the control and curbing of a mycotoxin known as patulin (PAT) in apple-derived beverages. PAT, g-lactone a-b unsaturated {4-hydroxy-4H-furol [3, 2-c] pyran-2(6H)-one}, is a secondary metabolite produced by *Penicillium* sp., molds that are present in certain foods, with *Penicillium expansum* being among the most prevalent and problematic sources of PAT [1]. Various treatments have been investigated for the ability to reduce the apple rot and PAT production within the harvest, processing, and storage steps. Control of postharvest pathogens still relies on the use of fungicides, but due to fungicide-resistant pathogens and public outcry for reduction and use of alternative control strategies, different treatment routes are considered. The legal and social constraints on the use of synthetic fungicides during fruit storage has led some researchers to investigate the use of natural food ingredients during apple storage. Reduction of mold using flume water during washing of apples after they have been harvested has often been considered [2]. To reduce the frequency of wounding during storage, a long-term-controlled atmosphere has now been shown to allow less stem-based fungal invasion into apples. There is conflicting evidence of whether or not controlled standard refrigeration is sufficient to prevent soft rot [3]. Several methods showing potential to control *P. expansum* and/or reduce patulin levels have been considered. These methods include the use of lemon and orange oil [4], and hydrogen peroxide [5]. Recently biological attempts show developments in the use of thiol-based yeast bio sorbents demonstrating efficiency in the removal of PAT [6]. PAT is a considerably resilient, heat-stable compound in aqueous acidic solutions at pH ranges of 3.5–5.5 which culminates in many problems associated with its removal during apple juice manufacture [7,8]. Additionally, more than 90 fungal species have been described to cause decay of apples during storage [9] thus contributing to PAT concentration in the final product. Often selections from salvage fruit, especially fruits that are unsuitable for slices and sauces, i.e. damaged by mechanical harvesting, windfall, insect-infestation, or culled fruit, are used to produce juice. These fruits with bruises, skin breaks, and other physical damage provide a perfect entry for PAT producing molds and their proliferation.

PAT ingested by humans poses detrimental health implications. In animal trials, PAT was found to cause ulceration and disruption of the gastrointestinal tract, by displacing Gram-positive bacteria; thereby, allowing the propagation of Gram-negative pathogens [10]. There have been 300+ different mycotoxins discovered by scientists as being present in food and feed since early in the history of humankind [11]. It is possible to determine which of these are important based on the frequency of occurrence and/or the severity of the diseases they cause, especially if they are known to be carcinogenic. Aflatoxin, deoxysorcinol, fumonisin, zearalenone, T-2 toxin, aspergillus toxin, and certain ergot alkaloids are amongst the most studied mycotoxins. The diseases (mycotoxicoses) caused by these mycotoxins are numerous and include susceptibility in animals and humans [12]. However, little evidence has been found of PAT being a carcinogen [13]. In rodents, PAT is said to disrupt fetal development [13,14]. All PAT-associated symptoms are inherently either directly or indirectly caused by the disruption of disulfide groups on proteins as a result of enzyme inhibition [15]. This means that humans can face challenges when ingesting beverages and foods laced with PAT.

Generally, PAT in apple juice and all apple-derived foods may not exceed 50 µg/L and 25 µg/L, respectively. European Union regulations 1425/3003 set a 50 µg/L limit (maximum) for fruit juices and 25 µg/L (maximum) for apple-derived products. The control of PAT in fruit juice is a challenging process that is interconnected with harvesting and post-processing procedures. There are several possible entry points for PAT and its distribution into the final products. Hence, the implementation of vigorous PAT control methods within the juice-manufacturing industry. Ultimately, the key to producing contaminant-free juices is consequent to the use of contaminant-free fruit, a challenging proposition. This situation might be possible at small-scale production to a certain degree but is not easy to implement for large scale industries. Consequently, studies into innovative methods for reduction, control, and monitoring of PAT prevalence are needed as the prevalence of PAT contamination in apple products is well recognized [15].

Over the last few decades, various methods of PAT removal have been explored, some with minimal success. These include filtering and adsorption, chemical modification, electromagnetic irradiation, and biological control [16]. The food-processing industry seeks a bio-friendly, non-thermal, and innovative way of treating PAT in fruit juice. There have been demonstrations of a vast number of methods and approaches to reduce PAT concentration in juice. A study has been done to demonstrate the reduction of PAT using H_2_O_2_ [5] and the use of photocatalysis using zinc oxide nanoparticles [17]. Recently, a study has been published for the treatment of apple juice using a novel adsorbent Fe_3_O_4_@SiO_2_@CS-GO@MIP, consisting a molecular imprinted polymer (MIP) prepared using a surface-imprinted technique coupled with Fe_3_O_4_, with magnetic properties assisting in the separation of the adsorbent from the food matrix, in combination with chitosan (CS) and SiO_2_ to improve biocompatibility and dispensability of the MIP [18]. However, there is no record of PAT treatment using a heterogeneous Fenton oxidation system applying nano zero-valent iron (nZVI) nanoparticles. Due to little focus on the application of nano-technological methods for the treatment of mycotoxins in food processing, this study proposes the use of photo-oxidation with nanomaterials. nZVI particles are commonly used in environmental remediation systems, typically in wastewater treatment [19], for the removal of pharmaceuticals, pesticides, and other organic contaminants [20]. The advantage of using nano-particles is associated with their large surface area which allows for good contact with the sample. Additionally, nano-particles have useful physicochemical properties owing to their quantum size; hence, they form an exceptional treatment support material [21]. Additionally, iron is a necessary nutritional element in acceptable doses; therefore, it is deemed safe for application in the treatment of PAT-contaminated juice. A common method for the synthesis of nano zero-valent metals is the chemical reduction of the metallic salts. In the synthesis of nZVI, sodium borohydrate (NaBH_4_) can be used as a reducing agent.

Methods that are not commonly in use but have the potential of being developed to be used due to their advantages are: precision milling, carbothermal reduction, ultrasound-assisted production, electrochemical generation, and green synthesis [22]. In this study, a green synthesis approach applying carbothermal reduction using olive-oil mill waste (OMW) as a carbon source, to produce encapsulated nano-zero valent iron (CE-nZVI) particles, was considered [23]. The selection of these particles is motivated by their potential ability to release fewer dissolved iron ions into the juice. In the treatment process, the nanoparticles supported the Fenton oxidation whereby a combination of nZVI in a liquid matrix and hydrogen peroxide (H_2_O_2_) known as a Fenton reagent promoted the generation of highly reactive hydroxyl radicals (•OH) which can oxidize numerous organic contaminants rapidly including PAT. There are various Fenton processes available for the decontamination of toxins. These include homogenous, heterogeneous, and photo-Fenton reactions [24].

This study was focused on the remediation of PAT from apple juice using a novel approach within fruit juice industry, i.e. heterogeneous Fenton oxidation of PAT using CE-nZVI f in apple juice for This involved introducing effective innovative methods for the elimination of PAT. The aim of using CE-nZVI was to guarantee the release of fewer iron ions in the PAT-treated juice with a possibility of the process developed being implementable by small-scale juicers.

## 2. Materials and Methods

### 2.1. Reagents for Analytical Methods and Procedures

Apple juice was purchased from a local grocery store (Alicante, Spain), while 5 mg (99%) PAT standard was purchased from Alfa Aesar (Kandel, Germany) with 5-Hydroxymethyl-2-furfural (HMF) being purchased as a 10 mg (97%) standard from Sigma Aldrich (Madrid, Spain). Similarly, other analytical grade reagents purchased from Sigma Aldrich (Madrid, Spain) were acetonitrile ethyl acetate, methanol, hexane, acetone, glacial acetic anhydrous sodium sulphate, sodium carbonate, and sodium acetate with microfiltered millipore quality water being obtained using a water deionizer L2, LAB-ION^®^. All other reagents used were also of an analytical grade standard.

### 2.2. Standards and Solution Preparation Procedures

Standard solutions of 5 mg/L of PAT and 10 mg/L HMF were dissolved to the desired concentrations in amber 1 L volumetric flasks. PAT standard solutions were wrapped in foil and were stored at temperatures of 2–5 °C. Working standard solutions of 5, 10, 50, 100, 200, and 400 µg/L of PAT were prepared. An HMF standard of 400 µg/L solution was prepared for comparative studies.

An acetate buffer (pH 4.0) was prepared by adding 0.45 mL acetic acid (glacial) to 40 mL of ultra-pure LAB-ION^®^ prepared H_2_O, subsequent to the dissolution of 0.1477 g CH_3_COONa in the acid solution, followed by adjusting the pH to 4.0 with acetic acid glacial. The volume was adjusted to 50 mL with ultra-pure LAB-ION^®^ prepared H_2_O after a pH titration procedure [25]. The buffer solution was stored in an amber bottle.

### 2.3. Solid-Phase Extraction (SEP)

The SEP method was adapted from Li et al. (2007). Specifically, the C18-SPE cartridges (Sigma Aldrich, Madrid, Spain) were pre-washed with 10 mL methanol, 3 mL (10%) methanol, and 10 mL water consecutively before use. The cartridges were not allowed to run dry. A volume of 5 mL for each sample containing 0.5 mL of the acetic acid buffer solution was transferred onto the column and allowed to percolate at 2 to 3 mL/min under a gentle suction. As soon as the solvent had drained, and the transfer was completed, the column walls were washed with 5 mL hexane and the column packing dried with a strong stream of air for 15 min. These eluents were discarded, and then the receiver was replaced by a small flask (with screw cap). The column was eluted with 5 mL analytical grade eluting solvents (hexane, ethyl acetate, acetone = 1:5:4, 1:4:5, 1:3:6, respectively), with the flow of each being stopped for approximately 1 min to allow the solvent sufficient contact time with the SPE column packing. Thereafter, one drop of glacial acetic acid was added to the combined solutions, followed by evaporation to dryness in a water bath at 40 °C under a gentle stream of nitrogen. The residue was immediately dissolved in 1 mL of the acetic acid buffer solution and injected into the HPLC system for analysis [25].

### 2.4. Reagents for the Hydrothermal Carbonization (HTC) of CE-nZVI

Olive mill waste (OMW) was locally sourced from an olive mill company from the Extremadura region in Spain, with Fe(NO_3_)_3_·9H_2_O (Sigma Aldrich, Madrid, Spain), ethanol (Alcohols Montplet S A, Barcelona, Spain) and 37% HCl (Fisher Scientific, Madrid, Spain) being purchased as analytical grade reagents from different suppliers as indicated. All solutions were prepared with ultra-pure LAB-ION^®^ prepared H_2_O.

### 2.5. Hydrothermal Carbonization (HTC) Synthesis of CE-nZVI

The OMW was clarified by centrifugation at 5000 rpm for 30 min using a 4K10 centrifuge (Sigma Aldrich, Madrid, Spain), followed by filtration with an 8 µm fiberglass filter based on work done by Calderon et al. (2018). In this experiment, the iron salt Fe(NO_3_)_3_·9H_2_O was reduced through a hydrothermal reaction. A volume of 300 mL of the OMW was mixed with 0.057 mol of Fe(NO_3_)_3_·9H_2_O for 1 h. The mixture was then transferred to an hydrothermal carbonization (HTC) reactor and was heated to 225 °C for 2 h. The pressure measured was 2780 kPA. The reactor was then left to cool overnight. The mixture was filtered under vacuum with a 0.2 µm cellulose acetate filter and washed twice with a 50:50 ethanol:water solution. The filtrate was placed in an oven at a temperature of 80 °C for 12 h. The sample was then ground into a fine powder and transferred to a vial for characterization. Thereafter, samples were treated at high temperatures (700 °C) under anaerobic conditions for annealing. Particles were inserted into a quartz tube in an oven for 3 h under nitrogen gas flow of 500 mL/min.

### 2.6. Experimental Set-Up for Heterogenous Fenton Oxidation Reactions

PAT degradation was carried-out using adsorption and heterogeneous Fenton oxidation in a four-necked 1 L Pyrex flask reactor vessel (Figure S1). The vessel was made up to 800 mL with the PAT-spiked solutions of either water or apple juice. The water matrix experiment reactions were conducted using a PAT concentration of 400 µg/L. The reactions were initiated by the introduction of active degradation components with concentrations of 0.05 g/L CE-nZVI and 0.5 mM H_2_O_2_. Initially, the reaction was undertaken at the solutions’ initial pH. Thereafter pH adjustments were made using 0.1 M NaOH and or 0.1 M HCl. Reactions were monitored over a 4-h period. At each measuring interval, a sample was drawn using a 5 mL syringe and filtered using a 0.45 µm filter. Thereafter, 10 µL of 1-butanol was added to the liquid samples to prohibit further oxidation by scavenging the action of the hydroxyl radicals.

### 2.7. Instrumentation and Working Parameters

An Agilent 1100 series HPLC (Agilent Technologies, Madrid, Spain) system was configured for PAT quantification. The analysis of a series of placebo matrix water samples and apple juice was conducted using liquid chromatography (LC) coupled with a degassing quaternary pump system. The chromatographic separation of analytes was achieved with a non-polar reversed-phase Sulpeco C18 (150 mm × 4 mm, 5 µm particle size, pore size 100 Å). An isothermal elution program was applied whereby a 10:90 acetonitrile: water mobile phase at a flow rate of 1 mL/min was used. The total run time was 7 min.

### 2.8. Inductively-Coupled Plasma-Mass Spectroscopy (ICP-MS)

Dissolved iron concentration was determined using ICP-MS using a Perkin Elmer Optima 4300 (Perkin Elmer, Madrid, Spain) DV (Dual Vision) after acidulating the samples with 2% of nitric acid.

### 2.9. Nanoparticle Characterization

Transmission electron microscopy (TEM) images were obtained with a Jeol Jem-2010 (Jeol, Freising, Germany) equipped with X-ray detector (Oxford INCA Energy TEM 100) for EDS microanalysis, to determine the size, aggregation, and composition of the nanoparticles. The Brunauer–Emmett–Teller (BET) surface area and pore size distribution (PSD) of CE-nZVI particles were obtained by physical adsorption using nitrogen at 77 K with an Autosob-6 (Qauntachrome Instruments, Madrid, Spain). The X-Ray Diffraction (XRD) analysis of CE-nZVI was performed to determine the crystalline structure of the nanoparticles. The equipment used was a Bruker D8-Advance with a CuKα radiation at 40 kV and a step size of 0.05 2θ at a 3 s/step.

## 3. Results and Discussion

### 3.1. HPLC Detection of PAT

**Figure 1:**
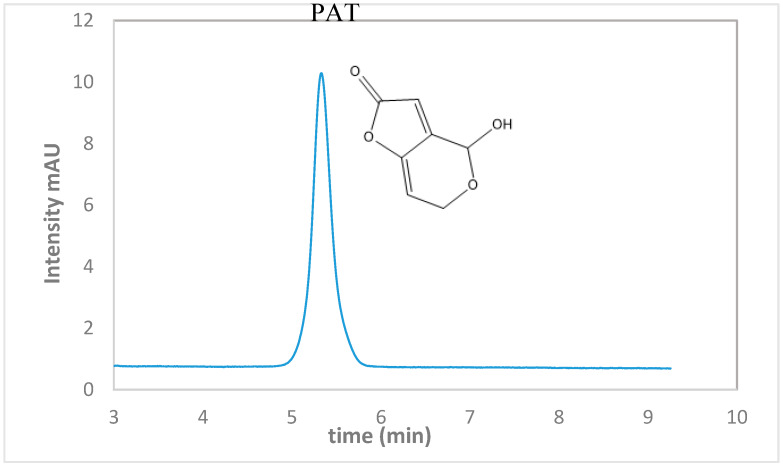
HPLC chromatogram of 400 µg/L patulin (PAT) standard (mAU: milli absorbance unit is absorbance intensity at 276 nm).

The initial concern in the remediation of PAT was a method displaying high sensitivity that would allow the detection of PAT at low concentrations of 5µg/L. This would enable investigation at low concentration levels of PAT upon degradation. The chromatogram obtained (Figure 1) for the analysis of 400 µg/L PAT standard, showed a distinct peak obtained at a retention time of 5.34 min with a total run time of 7 min with accurate concentration values. A calibration curve was conducted to ensure good linearity for concentration ranges between 5–400 µg/L. A correlation factor of 0.9595 was obtained. In order to validate the method, we assessed linearities, accuracies, repeatability, limit of detection (LOD), and limit of quantitation (LOQ), used to evaluate the extraction and cleanup. Thereafter LOQ of 4.89 µg/L were obtained for the target analytes; moreover, the method provided a low LOQ and LOD that was found to be 1.48 µg/L. Recoveries were determined at three fortification levels (50, 100, and 200 µg/L), with recoveries of 97.8%–98.9%, repeatability of relative standard deviation (RSD) of 0.61%, and reproducibility of RSDs not accounted for. Quality control criteria prescribed by the European Commission Directorate-General for Health and Food Safety guidelines (SANTE/12809/2016) were met during our investigation.

### 3.2. Carbothermal-Synthesized CE-nZVI

The material obtained from the production of the encapsulated nZVI at a temperature of 225 °C was composed of nanospheres, instead of microspheres of 6–8 µm which are usually obtained when glucose or sucrose are used as nanostructure synthesis reagents [26,27]. These particles presented had a higher surface area and greater incorporation of iron into the material. This is reported to be due to the different carbon sources used compared to the traditional refined carbon sources which are regularly used [23]. In CE-nZVI synthesis, there existed a diverse variety of polyphenols, organic acids, alcohols, and lipids in the hydrolysis and condensation reactions that formed the nanoparticles. The method undertaken with olive mill waste (OMW) is said to be complex as a result of there being none of the innate reactions that have been accounted for in literature. However, the product was related to the decomposed species generated from the starting materials used.

**Figure 2:**
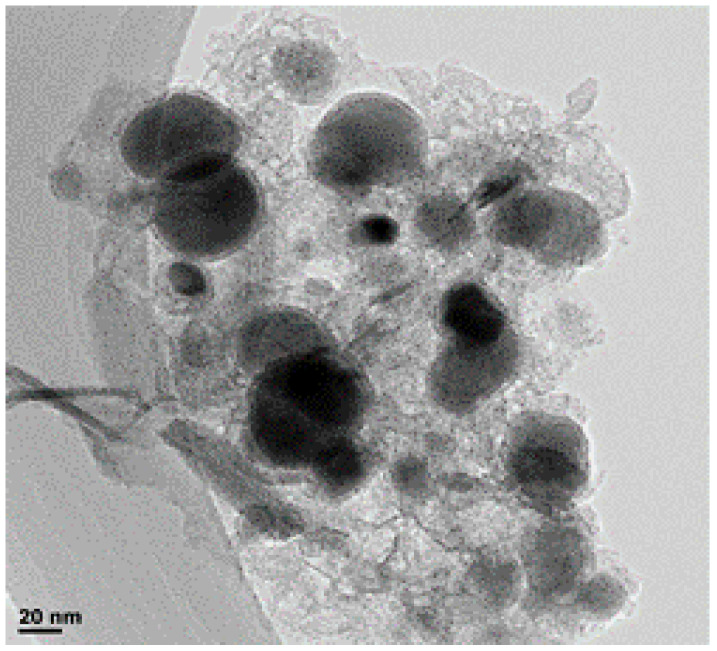
TEM images of carbon-encapsulated nano-zero valent iron (CE-nZVI) detailed structure of core-shell (on the lower left part of the image, the bar shown corresponds to a 20 nm distance in the picture).

TEM structures observed arising from the interaction of the electron beam with the composites showed that the CE-nZVI particles were spherical with sizes of 130 ± 50 nm (Figure 2). The BET surface area of the particles prepared over a period of 3 h was 190 m^2^/g.

### 3.3. Remediation of PAT Using CE-nZVI Coupled with a Fenton Reagent in Water Placebo Samples

Experiments were conducted for the remediation of PAT using a CE-nZVI load of 0.05 g/L to a solution inoculated with 400 µg/L of PAT and 0.5 mM H_2_O_2_ made up to a volume of 800 mL with deionized water. The chemistry of the Fe^0^–H_2_O_2_–H_2_O–PAT system was studied to resolve working conditions for the analysis of apple juice samples. Following CE-nZVI–PAT experiments, after a reaction period of 4 h, the percentage loss concentration obtained was 7.7%. Consequent to the low adsorption attempt in treating PAT using solely nanoparticles CE-nZVI, the study was directed toward a heterogenous Fenton oxidation approach. Molecular O_2_ and H_2_O_2_ are deemed ecologically-ideal oxidants and they are reduced in the presence of Fe aqua-ion compounds generating highly-reactive hydroxyl radicals and superoxide radicals as well as species of ferryl-type oxidants in the presence of H_2_O_2_. The oxidation process is an environmentally-benign method that contains chemicals that are non-toxic to humans.

The rapid reactivity of these reactants makes it difficult to trace their specific and non-specific activity and the influence of different parameters, i.e., temperature, nature of contaminant, ratio of composition of components, characteristic of oxidants, the concentration of the components, etc. This makes the studying of the mechanism for the catalytic process challenging. To establish the mechanism of the catalytic oxidation of various organic substances, it is important to have information about the radicals and non-radicals that are present, and about the intermediates formed under these conditions. Therefore, it is imperative to consider the conditions of the reactions. Henceforth, the effect of change in the pH on the radical-ion cyclic mechanism was considered. The initial experiment was conducted at a pH of 6 with the experiments observed over a 4-h period.

**Figure 3:**
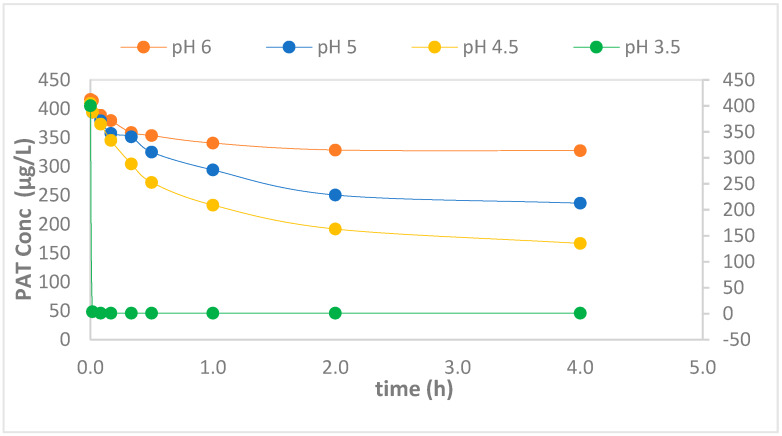
pH effect on heterogeneous Fenton reaction of CE-nZVI/H_2_O_2_.

Figure 3 shows the effect of initial pH on the degradation of PAT in the presence of CE-nZVI/H_2_O_2_ maintaining a FeO load of 0.05 g/L. As can be seen, starting reactions at different pH, influences the degradation of PAT. The removal of PAT was more significant at pH 3.5 and 4.5 in CE-nZVI/H_2_O_2_ systems. The percentage removal of PAT for CE-nZVI/H_2_O_2_ was found to be 99.75% and 66% at pH 3.5 and 4.5, respectively. In acidic conditions, the Fe^2+^/Fe^3+^ aqua ions generated from the oxidation of Fe^0^ are available and are kept in solution. Both are responsible for the catalytic consumption of H_2_O_2_ for the generation of hydroxyl radicals. In the case of pH 5 and pH 6, lower degradation below 50%, was observed. Babuponnusami and Muthukamar (2014) reported that Fenton reactions are strongly dependent on the pH of the solution and that pH 3 is the optimum pH for degradation of any target substrate due to the catalytic cycle of H_2_O_2_ and Fe^2+^ being favored [28]. At high pH, the corrosion of Fe^0^ is low thus there is less delivery of Fe^2+/^Fe^3+^, and consequently, less hydroxyl species formation for degradation. CE-nZVI/H_2_O_2_ showed good degradation; therefore, a concern that remained was the level of Fe^2+/^Fe^3+^ released into the solution or apple juice, if any.

**Figure 4:**
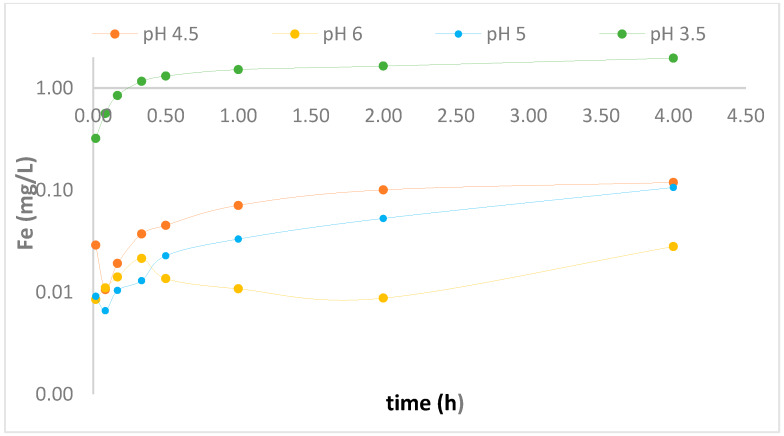
Release of dissolved iron ions -log Fe concentration (mg/L) during heterogeneous Fenton reaction of CE-nZVI/H_2_O_2_.

In turn, the results of dissolved iron ions released into the solution post-treatment were considered. This was a concern due to regulations passed on the limit of iron content in consumable water. The guidelines propose a health-based value of 2 mg/L which is higher than the acceptability threshold [29]. The results obtained showed that at the acidic medium pH 3.5, and after 1.0 min of treatment, the Fe concentration in solution was 0.99 mg/L with PAT removal from 400 to 3.61 mg/L, which is 99.1% degradation, whereas 99.75% degradation was observed at 4 h; albeit, a slightly higher concentration (1.64 mg/L) of dissolved Fe ions (Table S1), being released into solution this is still below proposed health-based value, as illustrated in Figure 4. Therefore, the use of CE-nZVI/H_2_O_2_ proved to be an effective method in the degradation of PAT and ensured a low concentration of dissolved Fe in solution. Apple juice samples treated at a pH of 3.4 indicated the pH condition is suitable for further exploration for mitigation of PAT in apple juices as discussed in the subsequent section.

To remediate PAT in apple juice samples, the heterogeneous Fenton oxidation (CE-nZVI/H_2_O_2_) system development was considered. However, the apple juice matrix is complex as it contains a mixture of sugars, primarily but not limited to fructose, glucose, and sucrose, including amides and other nitrogenous compounds and soluble pectin. Taking this into consideration, the treatment of PAT proved rather challenging due to the non-selective hydroxyl radical generated that randomly attacks any organic compounds within samples being treated. Therefore, a competitive environment for hydroxyl ions might affect the effectiveness of the oxidation of PAT. The presence of the diverse components also affects the HPLC trace chromatograph, with interfering compounds that are visible at the HPLC’s operating wavelengths. In the event of removing interfering compounds, the use of SPE extraction methods post-treatment was adopted. Treatment with CE-nZVI/H_2_O_2_ is a novel approach with regard to PAT remediation in apple juice. Overall, the method is inexpensive and involves harmless reactants such as Fe^0^ and H_2_O_2_. The Fenton reaction had proven to be successful at pH 3.5 for the treatment of PAT in a water matrix. Generally, the pH of the apple juice solution is between 3.35 and 4 [30]; therefore, pH adjustments were not necessary.

**Figure 5:**
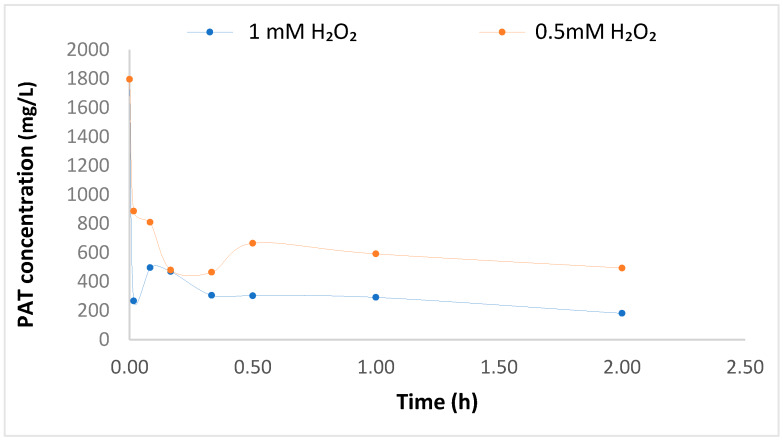
Graph illustrating degradation of PAT in apple juice sample for systems at different H_2_O_2_ concentrations.

**Figure 6:**
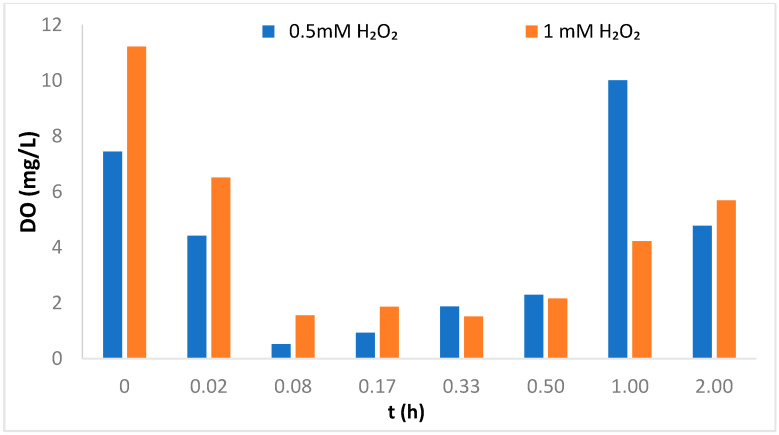
The concentration of DO (dissolved oxygen) during heterogeneous Fenton oxidation of juice sample.

The initial experiment was conducted using 0.5 mM H_2_O_2_, which showed a sharp decrease of PAT concentration after the introduction of CE-nZVI, at a Fe^0^ load of 0.05 g/L. Thereafter, a slight increase in PAT concentration was observed; followed by a plateau at the 30 min interval. Finally, at 2 h, the percentage of PAT degradation was found to be 72%. This allowed for a window to consider further investigations to ensure further degradation. PAT was then investigated at a higher H_2_O_2_ concentration of 1 mM – see Figure 5. Here, a high degradation at 1 min was followed by an increase in PAT concentration after 5 min. This was a repetitive phenomenon obtained in the initial experiment. After that, a plateau in the PAT concentration after 30 min was observed. Finally, after a 2-h reaction time, 89% degradation of PAT was achieved. In both experiments, the decrease in the initial PAT concentration may have been due to a surge of ferrous irons released upon O_2_ consumption (Figure 5) which produces a pulse of hydroxyl radicals in solution jointly with the adsorption of PAT. The decrease in the degradation may be allotted to possible surface passivation of CE-nZVI or possibly due to competing organic contaminants resulting in the desorption of PAT from some CE-nZVI. The regeneration of O_2_ (Figure 6) thereafter, was possibly influenced by the degradation associated with Fenton oxidation. A similar phenomenon has been reported in previous studies [31,32]. Additionally, studies done by Lee et al. (2008) further explained the limitation of the nZVI/O_2_ system and explored the use of Ni-Fe/O_2_ bimetallic systems to enhance contaminant degradation [33]. Chen et al. (2020) used FeS for the oxidation of phenol noting that at higher O_2_ content there is an acceleration in the degradation of phenol [34]. This further supports that the degradation phenomenon is related to the O_2_ distribution within the system. The results obtained at 2 h are a good indication of the potential of elimination of PAT using CE-nZVI/H_2_O_2_ in a heavily PAT-contaminated sample; albeit, the optimization of the degradation by increasing the concentration of the Fenton reagent can be explored. This can be considered as a clear remediation of PAT in apple juice.

## 4. Conclusions

In this study, CE-nZVI nanoparticles were synthesized using a thermal carbonization method and the particle size and surface area were measured to be 130 ± 50 nm and 190 m^2^/g in addition used for the heterogeneous Fenton degradation of patulin in water and apple juice. An HPLC method was modified and adapted for the measurement of patulin concentration after the treatments. Results observed showed an average percentage loss of 27.8%, 87%, 98%, and 99.75% at pH 6, 5, 4.5, and 3.5, respectively, during the monitoring period of 1 min to 4 h in the water matrix. Finally, the process was developed to focus on the remediation of PAT in commercially-available apple juice. The heterogeneous Fenton treatment showed a degradation trend for both 0.5 mM H_2_O_2_ and 1 mM H_2_O_2_ with PAT degradation of 72% and 89% respectively after 2-h treatment. Hence, the degradation efficiency of PAT using CE-nZVI and H_2_O_2_ showed to increase with decreasing pH and higher H_2_O_2_ dose. The heterogeneous Fenton oxidation method is a promising route for the degradation of PAT in apple juice.

## Supplementary Materials

The following are available online at https://www.mdpi.com/2304-8158/9/5/674/s1: Figure S1: Shows the reaction setup for Fenton oxidation system, Table S1: Concentration of dissolved iron ions at 4-h time interval.

## References

[1] Tangni E.K., Theys R., Mignolet E., Maudoux M., Michelet J.Y., Larondelle Y. (2003). Patulin in Domestic and Imported Apple-Based Drinks in Belgium: Occurrence and Exposure Assessment. Food Addit. Contam..

[2] Sydenham E.W., Vismer H.F., Marasas W.F., Brown N.L., Schlechter M., Rheeder J.P. (1997). The Influence of Deck Storage and Initial Processing on Patulin Levels in Apple Juice. Food Additves Contam..

[3] Paster N., Huppert D., Barkai Golan R. (1995). Production of Patulin by Different Strains of *Penicillium Expansum* in Pear and Apple Cultivars Stored at Different Temperatures and Modified Atmospheres. Food Addit. Contam..

[4] Hasan H.A.H. (2000). Patulin and Aflatoxin in Brown Rot Lesion of Apple Fruits and Their Regulation. World J. Microbiol. Biotechnol..

[5] Venturini M.E., Blanco D., Oria R. (2002). In Vitro Antifungal Activity of Several Antimicrobial Compounds against *Penicillium Expansum*. J. Food Prot..

[6] Qiu Y., Zhang Y., Wei J., Gu Y., Yue T., Yuan Y. (2020). Thiol-Functionalized Inactivated Yeast Embedded in Agar Aerogel for Highly Efficient Adsorption of Patulin in Apple Juice. J. Hazard. Mater..

[7] HARRISON M.A. (1988). Presence and Stability of Patulin in Apple Products: A Review. J. Food Saf..

[8] Biango-Daniels M.N., Snyder A.B., Worobo R.W., Hodge K.T. (2019). Fruit Infected with *Paecilomyces Niveus*: A Source of Spoilage Inoculum and Patulin in Apple Juice Concentrate?. Food Control.

[9] Leibinger W., Breuker B., Hahn M., Mendgen K. (1997). Control of Postharvest Pathogens and Colonization of the Apple Surface by Antagonistic Microorganisms in the Field. Phytopathology.

[10] Pal S., Singh N., Ansari K.M. (2017). Toxicological Effects of Patulin Mycotoxin on the Mammalian System: An Overview. Toxicol. Res. (Camb)..

[11] Wen J., Mu P., Deng Y. (2016). Mycotoxins: Cytotoxicity and Biotransformation in Animal Cells. Toxicol. Res..

[12] Richard J.L. (2007). Some Major Mycotoxins and Their Mycotoxicoses—An Overview. Int. J. Food Microbiol..

[13] Yiannikouris A., Jouany J.-P. (2002). Mycotoxins in Feeds and Their Fate in Animals: A Review. Anim. Res..

[14] Puel O., Galtier P., Oswald I.P. (2010). Biosynthesis and Toxicological Effects of Patulin. Toxins (Basel)..

[15] Ziarati P., Shirkhan F., Mostafidi M., Zahedi M.T., Sawicka B. (2019). Introduction of Methods to Reduce and Remove Patulin from Food Products. Bull. Univ. Agric. Sci. Vet. Med. Cluj-Napoca. Food Sci. Technol..

[16] Moake M.M., Padilla-Zakour O.I., Worobo R.W. (2005). Comprehensive Review of Patulin Control Methods in Foods. Compr. Rev. Food Sci. Food Saf..

[17] He L., Liu Y., Mustapha A., Lin M. (2011). Antifungal Activity of Zinc Oxide Nanoparticles against Botrytis Cinerea and Penicillium Expansum. Microbiol. Res..

[18] Sun J., Guo W., Ji J., Li Z., Yuan X., Pi F., Zhang Y., Sun X. (2019). Removal of Patulin in Apple Juice Based on Novel Magnetic Molecularly Imprinted Adsorbent Fe_3_O_4_@SiO2@CS-GO@MIP. LWT-Food Sci. Technol..

[19] Shu H.-Y., Chang M.-C., Chen C.-C., Chen P.-E. (2010). Using Resin Supported Nano Zero-Valent Iron Particles for Decoloration of Acid Blue 113 Azo Dye Solution. J. Hazard. Mater..

[20] Singh S.P., Bose P. Use of NZVI for Highly Persistence Chlorinated Pesticide DDT and Their Metabolites. Proceedings of the IWA World Congress on Water, Climate and Energy 2012.

[21] Mukherjee R., Kumar R., Sinha A., Lama Y., Saha A.K. (2016). A Review on Synthesis, Characterization, and Applications of Nano Zero Valent Iron (NZVI) for Environmental Remediation. Crit. Rev. Environ. Sci. Technol..

[22] Li L., Hu J., Shi X., Fan M., Luo J., Wei X. (2016). Nanoscale Zero-Valent Metals: A Review of Synthesis, Characterization, and Applications to Environmental Remediation. Environ. Sci. Pollut. Res..

[23] Calderon B., Smith F., Aracil I., Fullana A. (2018). Green Synthesis of Thin Shell Carbon-Encapsulated Iron Nanoparticles via Hydrothermal Carbonization. ACS Sustain. Chem. Eng..

[24] Litter M.I., Slodowicz M. (2017). An Overview on Heterogeneous Fenton and PhotoFenton Reactions Using Zerovalent Iron Materials. J. Adv. Oxid. Technol..

[25] Li J., Wu R., Hu Q., Wang J. (2007). Solid-Phase Extraction and HPLC Determination of Patulin in Apple Juice Concentrate. Food Control.

[26] Huang C.-C., Chen C.-H., Chu S.-M. (2006). Effect of Moisture on H2S Adsorption by Copper Impregnated Activated Carbon. J. Hazard. Mater..

[27] Wang Y., Sun H., Duan X., Ang H.M., Tadé M.O., Wang S. (2015). A New Magnetic Nano Zero-Valent Iron Encapsulated in Carbon Spheres for Oxidative Degradation of Phenol. Appl. Catal. B Environ..

[28] Babuponnusami A., Muthukumar K. (2014). A Review on Fenton and Improvements to the Fenton Process for Wastewater Treatment. J. Environ. Chem. Eng..

[29] WHO-EU (2017). Drinking Water Parameter Cooperation Project.

[30] US FDA/CFSAN-Approximate pH of Foods and Food Products. https://webpal.org/SAFE/aaarecovery/2_food_storage/Processing/lacf-phs.htm.

[31] Feitz A.J., Joo S.H., Guan J., Sun Q., Sedlak D.L., David Waite T. (2005). Oxidative Transformation of Contaminants Using Colloidal Zero-Valent Iron. Colloids Surfaces A Physicochem. Eng. Asp..

[32] Joo S.H., Feitz A.J., Waite T.D. (2004). Oxidative Degradation of the Carbothioate Herbicide, Molinate, Using Nanoscale Zero-Valent Iron. Environ. Sci. Technol..

[33] Lee C., Sedlak D.L. (2008). Enhanced Formation of Oxidants from Bimetallic Nickel-Iron Nanoparticles in the Presence of Oxygen. Environ. Sci. Technol..

[34] Cheng D., Neumann A., Yuan S., Liao W., Qian A. (2020). Oxidative Degradation of Organic Contaminants by FeS in the Presence of O2. Environ. Sci. Technol..

