# Supplementary material for: Heterogeneous Fenton Degradation of Patulin in Apple Juice Using Carbon-Encapsulated Nano Zero-Valent Iron (CE-nZVI)
Source: Foods. 2020 May 24;9(5):674. doi: 10.3390/foods9050674 (PMC7278583; doi:10.3390/foods9050674)
Supplement: Supplementary file 1 [file foods-09-00674-s001.pdf]

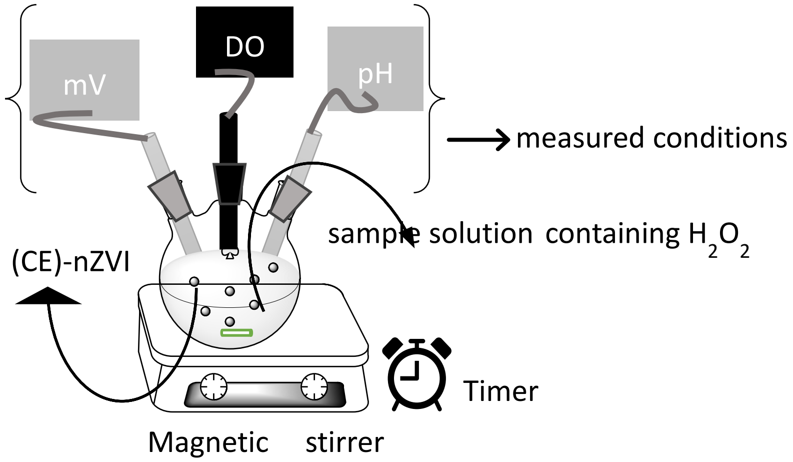

Figure S1. Shows the reaction setup for Fenton oxidation system

Table S1. Concentration of dissolved iron ions at 4-hour time interval.

**pH**	**Fe^{2+}/Fe^{3+} mg/l**
3.5	1.64
4.5	0.01
5	0.05
6	0.10
